# Ablative-Transarterial Radioembolization resulting in complete histopathological response of hepatocellular carcinoma in the resected liver specimen after salvage hepatectomy

**DOI:** 10.1016/j.ijscr.2021.106679

**Published:** 2021-12-28

**Authors:** Thomas Kam-Man Chung, Thomas Wai-Tong Leung, Cheuk-Hei Chung, Howard Ho-Wai Leung, Wan Yee Lau

**Affiliations:** a Department of Surgery, Prince of Wales Hospital, the Chinese University of Hong Kong, Hong Kong, China; b Comprehensive Oncology Center, Hong Kong Sanatorium of Hospital, 2 Village Road, Happy Valley, Hong Kong, China; c Department of Surgery, Princess Margaret Hospital, Hong Kong; d Department of Anatomical and Cellular Pathology, Prince of Wales Hospital, The Chinese University of Hong Kong, Hong Kong, China; e Faculty of Medicine, The Chinese University of Hong Kong, Shatin, Hong Kong, China

**Keywords:** Ablative-transarterial radioembolization (A-TARE), Hepatocellular carcinoma, Salvage hepatectomy

## Abstract

**Introduction:**

Hepatocellular carcinoma (HCC) is a common disease. Many patients at the time of diagnosis of HCC are in advanced stages and cannot benefit from curative treatment. Palliative treatments remain the only treatment option. Advances in palliative treatment can occasionally downstage HCC and induce enough liver hypertrophy to allow salvage hepatectomy to be performed on patients with initially unresectable HCC. We herein present a patient who underwent salvage hepatectomy after successful Ablative-Transarterial Radioembolization (A-TARE) with complete histopathologic response in the resected liver specimen.

**Case report:**

A 67-year old obese patient presented with a 9.7 cm HCC at liver segment 8, with local tumour extension to involve segments 4,5 and 7. Initial workup suggested the tumour to be unresectable. A-TARE with yttrium-90 microspheres was given. Further workup 4 months after A-TARE showed the tumour to be downstaged with adequate hypertrophy of future liver remnant. Salvage hepatectomy became possible and the patient underwent salvage trisectionectomy 5 months after A-TARE. He recovered uneventfully from the operation. Histopathological examination of the resected liver specimen showed no viable tumour cells inside a fibrous mass which corresponded to the radiologic residual tumour.

**Discussion:**

Salvage hepatectomy should be offered to patients after tumour downstaging with A-TARE as viable malignant cells are likely to persist. Complete response with no viable tumour cells in the resected liver specimen, to our knowledge, has never been reported in literature.

**Conclusion:**

A-TARE was able to induce complete histopathological response in a patient who initially presented with a large and unresectable HCC mass.

## Highlights

• Many patients at the time of diagnosis of HCC are in advanced stages and cannot benefit from curative treatment.
• Palliative treatments remain the only treatment option.
• Advances in palliative treatment can occasionally downstage HCC and induce enough liver hypertrophy to allow salvaged hepatectomy to be performed on patients with initially unresectable HCC.
• We herein present a 67-year old patient who underwent salvage hepatectomy after successful Ablative-Transarterial Radioembolization (A-TARE) with complete histopathologic response in the resected liver specimen.
• Complete response with no viable tumour cells in the resected liver specimen, to our knowledge, has never been reported in literature.

## 1 Introduction

Primary liver cancer is the sixth commonest cancer and the second leading cause of cancer-related death around the world. Hepatocellular carcinoma (HCC) is the commonest and predominant primary liver cancer in East Asia. Most patients with HCC are diagnosed at an advanced stage [1]. Only around 35% of patients at diagnosis are amenable to curative treatment [2]. Palliative treatments remain the only treatment-option for these patients, including transarterial chemoembolization (TACE), transarterial radioembolization (TARE), stereotactic body radiation therapy (SBRT) and systemic therapy [3]. Advances in some of these treatments can effectively shrink tumours and induce liver hypertrophy in future liver remnants (FLR) in a proportion of patients, resulting in tumour-downstaging from unresectable tumours to resectable, non-transplantable to transplantable. Salvage hepatectomy or liver transplantation then offers possible cure to these patients with initially non-curable HCC [4]. A patient who had an initially unresectable HCC which became successfully downstaged by Ablative-TARE (A-TARE) [5] using yttrium-90 microspheres followed-by salvage hepatectomy, and with complete histopathological response is herein reported.

## 2 Presentation of case

The patient was a 67-year-old obese gentleman with a history of hypertension and non-insulin dependent diabetes mellitus. His body mass index was 34.9. He was a non-smoker but a heavy alcohol drinker who stopped drinking only after diagnosis of HCC. His alcoholic liver cirrhosis was regularly followed-up in a public hospital. His serum serology for both hepatitis B and C was negative.

Six months prior to seeing us, a routine CT scan for follow-up on his cirrhosis showed a mass in segment 8 of the liver, measuring 6.6 cm. According to the radiologist, the diagnosis was suggestive of a benign focal nodular hyperplasia because the lesion had a central scar. The serum AFP was normal and the patient was asymptomatic. The managing physician decided to observe the lesion.

A follow-up PET CT scan 6 months later showed the right liver mass had increased in size to 9.7 × 7.2x8cm, and it had extended from liver segment 8 to involve part of segments 4,5 and 7. There was intense C11-acetate uptake suggesting the lesion to be a well-differentiated HCC. There was no portal vein tumour thrombosis and no extrahepatic metastasis. An incidental left upper eyelid subcutaneous nodule was excised and pathology showed moderately differentiated squamous cell carcinoma.

The patient was then further worked-up for liver surgery. The indocyanine green retention test at 15 min (ICG-R15) was 15.8%. The overall assessment suggested the patient was not a candidate for surgical resection because of inadequate volume of FLR and a high ICG.R15 test result. The international normalized ratio (INR) was 1.1 and platelet count was 138 10^9^/L. Liver function tests were normal with a Child-Pugh A liver function.

The patient came to consult us for a second surgical opinion. Due to tumour unresectability, he was suggested to undergo yttrium-90 microsphere therapy using a new technique called Ablative-Transarterial Radioembolization (A-TARE) [5].

**Fig. 1:**
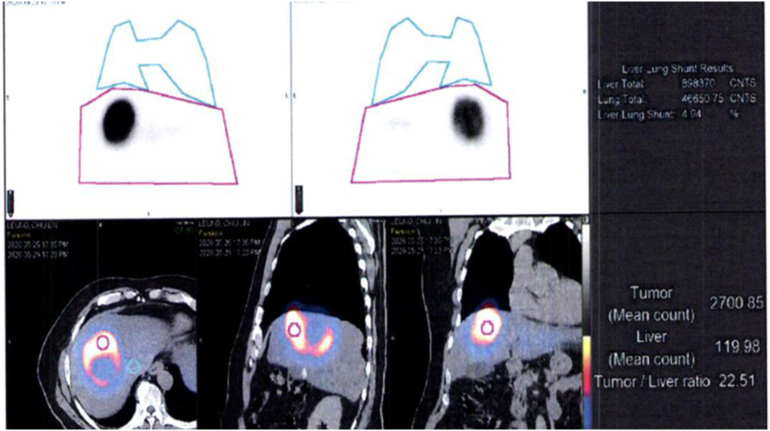
Tc MAA spectroscopy before ablative – TARE.

A mapping intra-arterial technetium labeled macroaggregated albumin scan (Tc-MAA) was performed in the work-up for A-TARE. Hepatic angiography showed a large and markedly hypervascular mass, measuring 9 cm, in the region of liver segments 4, 5, 7 and 8. The tumour was predominantly supplied by the right and partly by the left hepatic arteries. Two separate activities of Technetium macroaggregated albumin (Tc-MAA) were given through the right and left hepatic arteries respectively. Tc-MAA spectroscopy showed significant Tc-MAA accumulation within the tumour. A tumour to normal uptake ratio was 22.5. The lung shunting was 4.9% (Fig. 1).

About 10 days after complete work-up of A-TARE, he was treated with 2 separate activities of Therasphere (yttrium-90 glass microspheres by BTG, International Group Co., London), 3.48 GBq through the right hepatic artery and 0.55 GBq through the left hepatic artery. Post-treatment day 1 PET scan showed successful accumulation of yttrium-90 microspheres to the liver tumour. The distribution of yttrium-90 activity highly matched the Tc-MAA avid tumour uptakes. He developed no side effects to treatment and was discharged home on day 1 after the procedure.

Dosimetric calculations showed the tumour dose was up to 350Gy and the non-tumourous liver dose was below 7Gy. Post treatment showed stable liver function tests and no hepatic toxicity. The patient remained asymptomatic. He had a reassessment PET CT scan 2 months after A-TARE. The previously noted markedly C11 acetate avid mass in liver became smaller and less active, and showed no abnormal 18F-fluorodeoxyglucose (FDG) metabolism, suggestive of partial response to treatment. There was no extrahepatic metastasis.

**Fig. 2:**
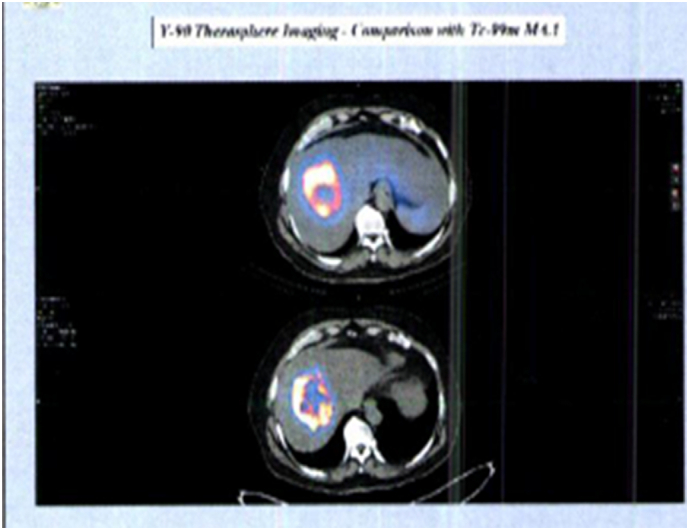
Accumulation of yttrium-90 microspheres in tumour as from PET scan on day 1 after treatment.

At 4 months after A-TARE, his liver function remained stable. CT scan showed hypertrophy of the non-tumourous liver in the FLR in liver segments 1, 2 and 3, and a shrunken tumour measuring 4.0 × 4.4 × 4.9 cm (Fig. 2). Measurement of segments 2 and 3 volume was 382.8 ml and segment 1 was 18.7 ml. The total liver volume was 1644 ml. The predicted FLR volume after right trisectionectomy was adequate. PET-CT scan showed no metastatic disease. The complete blood count, liver and renal function, and INR were all normal. ICG-R15 test was 5%.

**Fig. 3:**
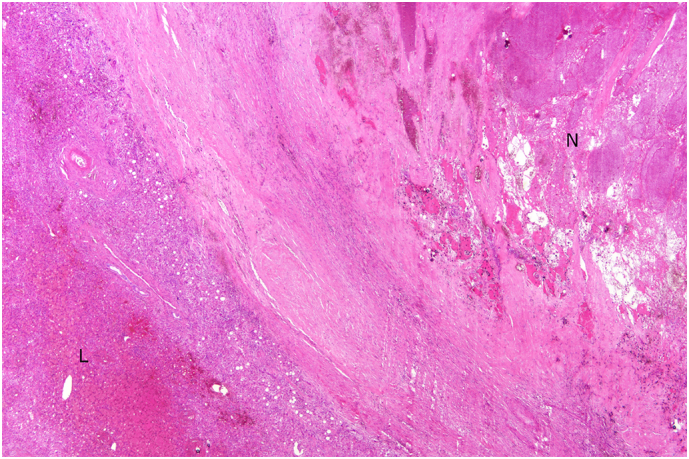
(H&E, x20) Necrotic nodule (N) and adjacent liver parenchyma (L) interface.

**Fig. 4:**
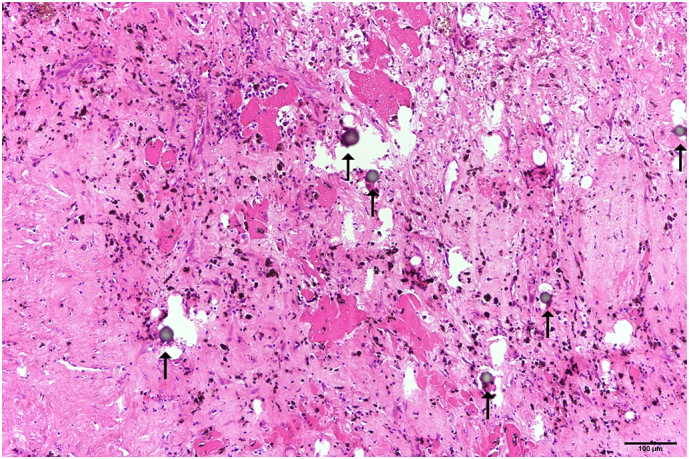
(H&E, x100) The nodule consists of hemorrhage, inflamed granulation tissue, hemosiderin depositions, and therapeutic emboli (arrows) with necrosis.

Right trisectionectomy was performed 5 months after A-TARE. The resected specimen on histopathological examination showed it to measure 15x18x6 cm, with a nodule of 4.5 cm located at 1.3 cm from the closest resection margin. Section of the nodule showed only fibrotic tissues with hemorrhage and no viable tumour cells (Fig. 3). Therapeutic emboli were noted in the nodule (Fig. 4). No tumour satellite lesions were detected. The patient recovered uneventfully from the operation. Post-operative CT scan showed a healthy liver remnant with good perfusion and no residual viable tumour. The patient was well with no evidence of tumour recurrence on follow-up.

## 3 Discussion

The diagnosis of HCC can be established without histology by detection of a liver mass with an elevated serum alpha fetoprotein of over 400 ng/ml in high risks patients. Alternatively, the diagnosis of HCC can be established by the 2010 American Association for the Study of Liver Disease (AASLD) guidelines [5] by using typical enhancement patterns on multi-detector row computed tomography (MDCT) [6]. Also, a substantial lesion growth, defined as an increase in its longest diameter by more than 5 mm in 12 months, is also diagnostic of HCC [6]. In our patient, a diagnosis of HCC was certain because of the radiologic appearances of the lesion on CT scan and dual tracer PET-CT scan, with an increase in size of more than 3 cm in 6 months. In retrospect, a lesion of >6 cm in diameter in a patient with a risk factor of developing HCC (alcoholic) should raise the suspicion of HCC even if the radiologic features favoured benign focal nodular hyperplasia. A more vigilant monitoring by medical imagings within a short duration should have been performed.

HCC derives its blood supply mainly from hepatic arteries, while 70–80% of blood supply to normal hepatic tissues from portal veins. TARE capitalizes this concept in delivering a high dosage of internal radiation to HCC with minimal dosage to non-tumourous liver parenchyma. Glass beads of size 20-30 μm which contain radioactive yttrium-90 have been shown to be effective in treating inoperable HCC. This technique has further evolved from non-selective internal radiation to the whole liver, to selective internal radiation to hemilivers/liver sections/liver segments, to high treatment radiation doses to hemilivers/liver sections/segments called radiation hepatectomy [7], and then to very high ablative treatment doses to HCC called ablative-TARE [5]. These latter developments give a chance of cure to patients who are not candidates for surgical resection because of severe co-morbidities. The observation of inducing hypertrophy of FLR and effective shrinkage of tumour in a patient with good liver function renders the concept of salvage hepatectomy a plausible option for previously unresectable HCC [8]. Furthermore, for patients with initially poor liver function with HCC which exceeds the Milan criteria for liver transplantation, tumour downstaging can convert a non-transplantable HCC into a transplantable one. Thus, patients with non-curable diseases can be downstaged and converted to become curable.

To our knowledge, this is the first case report on A-TARE which resulted in complete histopathological response of HCC as shown in the resected liver specimen after salvage hepatectomy. Thus, for patients who are not candidates for salvage hepatectomy because of co-morbidities, there is still a chance of cure for them to undergo A-TARE.

**Fig. 5:**
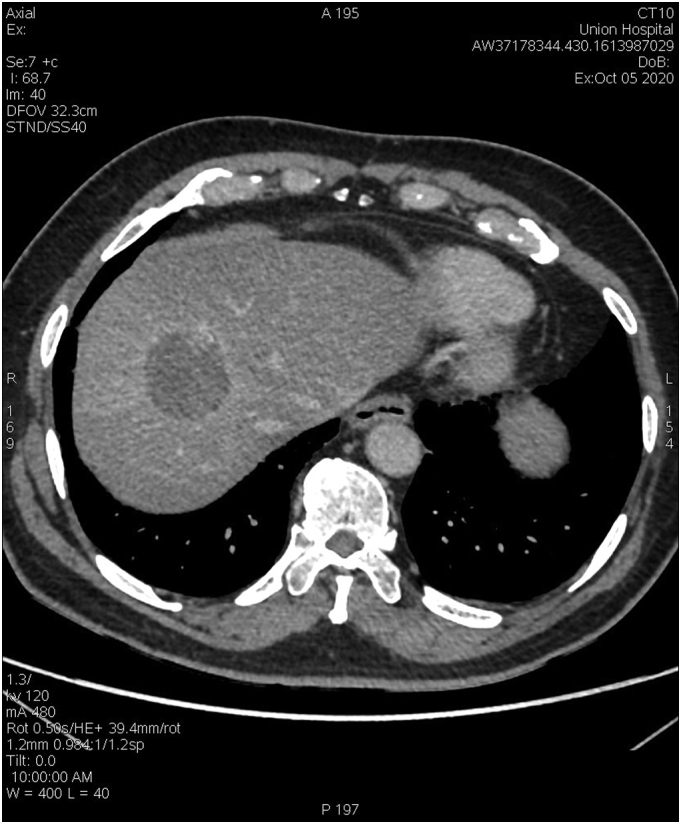
pre-operative CT scan, no visible middle hepatic vein was shown.

In this patient, salvage hepatectomy was technically challenging as the normal liver was distorted by A-TARE. Involvement of the middle hepatic vein and the shrunken right hemiliver made right trisectionectomy the only surgical option (Fig. 5). The hypertrophied left lateral section had enough volume and function to support this major liver resection. As a great proportion of patients showed viable tumour cells in the resected specimens after tumour downstaging and salvage liver resection or transplantation [8], the complete histopathological response to A-TARE came as a surprise to us.

## 4 Conclusions

A-TARE can be used to treat patients with unresectable HCC. Effective downstaging of the tumour with adequate hypertrophy of non-tumourous liver in this case report rendered salvage hepatectomy to become possible. A complete histopathological response found on the resected liver specimen after A-TARE came as a surprise to us. Such a good response suggested to us that A-TARE can result in possible cure to patients who have unreasectable HCC or who are not fit for liver resection because of severe associated co-morbidities.

## Funding

The authors received no financial support for publication of this case report.

## Consent

Written informed consent was obtained from this patient before writing up this manuscript.

## Registration of research studies

None.

## Provenance and peer review

Editorially reviewed, not externally peer-reviewed.

## Ethical approval

There is no need for ethical approval as this case report is on an excellent response which has not been reported to a reported procedure.

## Guarantor

Kam-Man Thomas Chung, first author of this article.

## CRediT authorship contribution statement

Wan Yee Lau, Kam-Man Thomas Chung: surgeons operating on the patient

Wai-Tong Thomas Leung: Oncologist performing yttrium-90 microsphere therapy

Wan Yee Lau: study concept, design, final proofreading

Kam-Man Thomas Chung, Wai-Tong Thomas Leung: preparation of manuscript

Cheuk-Hei Hilary Chung: literature search, preparation of manuscript, computer support and

electronic submission

Ho-Wai Howard Leung: Pathologist performing histologic examination of specimen, provision of histologic image for publication

## Declaration of competing interest

The authors report no declaration of interest.
